# Psychotic symptoms and Parkinson’s Disease: Clinical and Therapeutic Aspects

**DOI:** 10.1192/j.eurpsy.2022.1194

**Published:** 2022-09-01

**Authors:** D. Nzamba Nzamba, K. Benallel, A. Kaddaf, M. Gartoum, M. Kadiri

**Affiliations:** Military Teaching Hospital Mohammed V-Rabat, Department Of Psychiatry, Rabat, Morocco

**Keywords:** Psychosis, Therapeutic, Parkinson’s disease

## Abstract

**Introduction:**

Parkinson’s disease (PD), the second most common neurodegenerative disease after Alzheimer’s disease, affects 1% of the population after the age of 60. Motor symptoms are the most common features that may be associated with non-motor symptoms including psychotic symptoms.

**Objectives:**

Faire le point sur les modalités de prise en charge des symptômes psychotiques au cours de la maladie de Parkinson

**Methods:**

Nous décrivons 3 cas de développement de symptômes psychotiques, survenus chez des patients atteints de la maladie de Parkinson, et faisons le point sur la prise en charge des manifestations symptomatiques psychiatriques dans la maladie de Parkinson, par une brève revue de la littérature.

**Results:**

Case 1: 42-year-old man, with 5 years’ history of PD, presented with auditory hallucinations comorbid with paranoid personality disorder, which occurred 12 months following antiparkinsonian drugs use. Case 2: 58-year-old man, with 17 years’ history of PD, presented jealousy delusions and behavioral disorders, which occurred 12 years following antiparkinsonian drugs use. Case 3: 76-year-old man, with 36 years’ history of PD, presented visual hallucinations, subjective sensation of a presence and jealousy delusion, which occurred 26 years following antiparkinsonian drugs use.

**Conclusions:**

Les symptômes psychotiques de la maladie de Parkinson sont fréquents. La prise en charge consiste à traiter les symptômes psychotiques sans aggraver les symptômes moteurs liés à l’hypo-dopaminergie.

**Disclosure:**

No significant relationships.

